# Supplementary material for: Identification of photoperiod-induced specific miRNAs in the adrenal glands of Sunite sheep (Ovis aries)
Source: Front Vet Sci. 2022 Jul 22;9:888207. doi: 10.3389/fvets.2022.888207 (PMC9354845; doi:10.3389/fvets.2022.888207)
Supplement: Supplementary file 1 [file Data_Sheet_1.ZIP › Supplementary file/miRDeep2 file.zip!/miRDeep2 file/Know_Structure/SP42A2/oar-mir-221.pdf]

miRBase precursor : oar-mir-221
Total read count : 3376
oar-miR-221 read count : 3062
remaining reads : 314

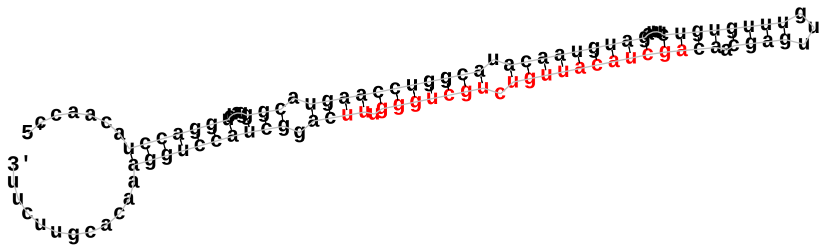

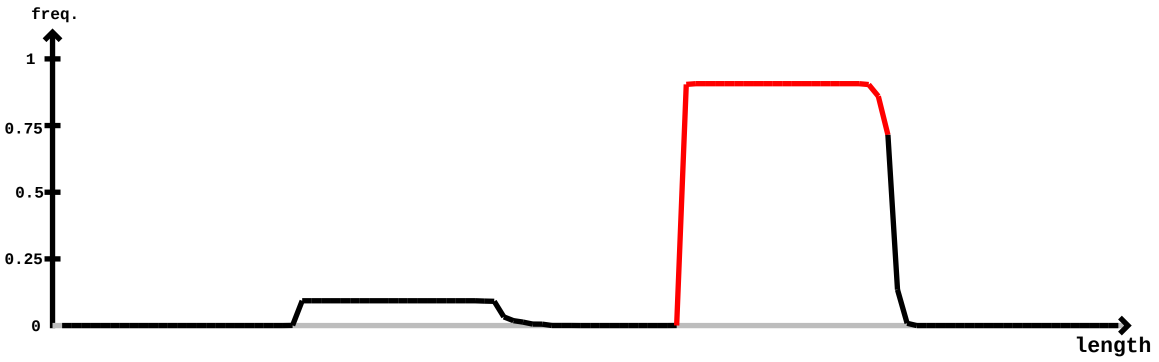

oar-miR-221

```
5'- ccaacauccaggucuagggcaugaaccuggcauacaauguagauuucuguguuuguugagcaacagcuacauugucugcuggguuucaggcuaccuggaaacacguucuu   -3'    exp
    ......(((((((....(((.(((((((((((.(((((((((....((((((((...)))).))))))))))))).))))))).)))).))))))))))...........   reads    mm    sample
    .......................aaccuggcauacaauguagauuucugu............................................................   2        0     seq
    ........................accuggcauacaauguaga...................................................................   4        0     seq
    ........................accuggcauacaauguagau..................................................................   3        0     seq
    ........................accuggcauacaauguagauu.................................................................   197      0     seq
    ........................accuggcauacaauguagauuu................................................................   48       0     seq
    ........................accuggcauacaauguagauuuc...............................................................   18       0     seq
    ........................accuggcauacaauguagauuucu..............................................................   25       0     seq
    ........................accuggcauacaauguagauuucug.............................................................   1        0     seq
    ........................accuggcauacaauguagauuucugu............................................................   15       0     seq
    ........................accuggcauacaauguagauuucuguguu.........................................................   1        0     seq
    ...............................................................cagcuacauugucugcuggguuu........................   1        0     seq
    ................................................................agcuacauugucugcuggg...........................   11       0     seq
    ................................................................agcuacauugucugcugggu..........................   148      0     seq
    ................................................................agcuacauugucugcuggguu.........................   486      0     seq
    ................................................................agcuacauugucugcuggguuu........................   1953     0     seq
    ................................................................agcuacauugucugcuggguuuc.......................   425      0     seq
    ................................................................agcuacauugucugcuggguuuca......................   29       0     seq
    .................................................................gcuacauugucugcuggguu.........................   2        0     seq
    .................................................................gcuacauugucugcuggguuu........................   7        0     seq
```
